# Association of non-invasive atrial cardiomyopathy markers with cerebral stroke lesions: a population-based analysis from the Hamburg City Health Study

**DOI:** 10.1093/europace/euag063

**Published:** 2026-03-27

**Authors:** Marie Biedermann, Lena Koch, Silvia Becker, Ana Bošnjak, Amir S Jadidi, Dirk Westermann, Heiko Lehrmann, Thomas Arentz, Axel Loewe, Bastian Cheng, Götz Thomalla, Helge Kniep, Jens Fiehler, Daniel Engler, Renate B Schnabel, Martin Eichenlaub

**Affiliations:** Department of Cardiology and Angiology, Medical Center, Faculty of Medicine, University of Freiburg, Suedring 15, Bad Krozingen 79189, Germany; Department of Cardiology and Angiology, Medical Center, Faculty of Medicine, University of Freiburg, Suedring 15, Bad Krozingen 79189, Germany; Department of Cardiology and Angiology, Medical Center, Faculty of Medicine, University of Freiburg, Suedring 15, Bad Krozingen 79189, Germany; Institute of Biomedical Engineering, Karlsruhe Institute of Technology (KIT), Karlsruhe, Germany; Department of Cardiology and Angiology, Medical Center, Faculty of Medicine, University of Freiburg, Suedring 15, Bad Krozingen 79189, Germany; Department of Cardiology, Heart Center Lucerne—Lucerne Cantonal Hospital, Lucerne, Switzerland; Department of Cardiology and Angiology, Medical Center, Faculty of Medicine, University of Freiburg, Suedring 15, Bad Krozingen 79189, Germany; Department of Cardiology and Angiology, Medical Center, Faculty of Medicine, University of Freiburg, Suedring 15, Bad Krozingen 79189, Germany; Department of Cardiology and Angiology, Medical Center, Faculty of Medicine, University of Freiburg, Suedring 15, Bad Krozingen 79189, Germany; Institute of Biomedical Engineering, Karlsruhe Institute of Technology (KIT), Karlsruhe, Germany; Department of Neurology, University Medical Center Hamburg-Eppendorf, Hamburg, Germany; Department of Neurology, University Medical Center Hamburg-Eppendorf, Hamburg, Germany; Department of Diagnostic and Interventional Neuroradiology, University Medical Center Hamburg-Eppendorf, Hamburg, Germany; Department of Diagnostic and Interventional Neuroradiology, University Medical Center Hamburg-Eppendorf, Hamburg, Germany; Department of Cardiology, University Heart and Vascular Center Hamburg, University Medical Center Hamburg-Eppendorf, Hamburg, Germany; German Center for Cardiovascular Research (DZHK), Partner Site Hamburg/Kiel/Lübeck, Hamburg, Germany; Department of Cardiology, University Heart and Vascular Center Hamburg, University Medical Center Hamburg-Eppendorf, Hamburg, Germany; German Center for Cardiovascular Research (DZHK), Partner Site Hamburg/Kiel/Lübeck, Hamburg, Germany; Department of Cardiology and Angiology, Medical Center, Faculty of Medicine, University of Freiburg, Suedring 15, Bad Krozingen 79189, Germany

**Keywords:** Stroke, Atrial fibrillation, Non-invasive screening, ECG, TTE, Biomarkers

## Abstract

**Aims:**

Atrial fibrillation (AF) is a well-known risk factor for ischaemic stroke. Emerging evidence suggests that atrial cardiomyopathy (AtCM), independent of AF, may be a key contributor to stroke risk. We aimed to evaluate whether non-invasive AtCM markers assessed by electrocardiography (ECG), transthoracic echocardiography (TTE), and blood-based biomarkers provide incremental diagnostic value beyond established clinical risk factors for identifying cerebral stroke lesions on magnetic resonance imaging (MRI).

**Methods and results:**

We analysed 1794 Hamburg City Health Study participants who underwent cerebral MRI with available baseline 12-lead ECG and TTE in sinus rhythm. Logistic regression analyses were performed to identify associations between non-invasive AtCM markers and stroke lesions. The incremental discriminatory performance of these markers beyond clinical risk factors was assessed. Stroke lesions were present in 152 participants (8.5%). Male sex, history of prior AF, and higher CHA_2_DS_2_-VA score were significantly associated with stroke lesions. Among AtCM markers, amplified P-wave duration (APWD), P-wave area in lead II, PR interval, left atrial volume index, left atrial ejection fraction, and NT-proBNP were also significant. Combining both clinical and AtCM markers, only CHA_2_DS_2_-VA score (OR: 1.95 per point, 95% CI: 1.49–2.55, *P* < 0.001) and P-wave area in lead II (OR: 0.99 per 100 µV·ms, 95% CI: 0.98–1.00, *P* = 0.031) remained independent predictors. However, this resulted in only marginal improvement in discrimination (ΔAUC: 0.03, 95% CI: 0.0001–0.0594) compared with the clinical risk factor model alone.

**Conclusion:**

The incremental diagnostic value of AtCM markers beyond clinical risk factors for MRI-defined cerebral lesions is limited, likely reflecting the heterogeneous aetiology of stroke lesions in a general population cohort.

## Graphical Abstract

**Graphical Abstract:**
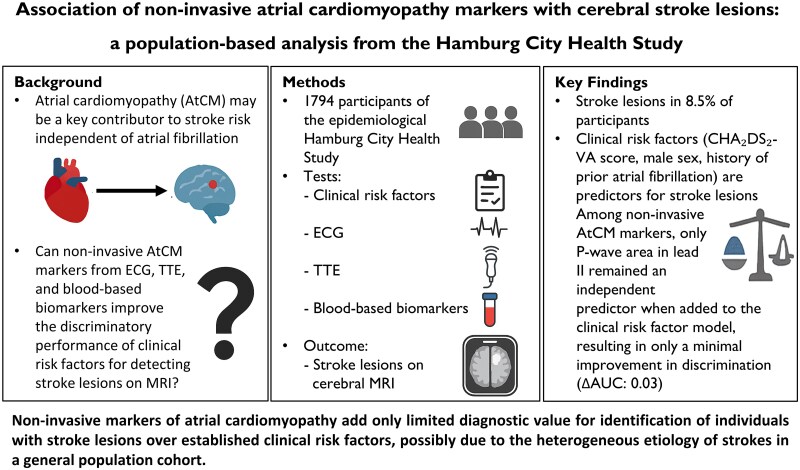

What’s new?

- This study provides a population-based evaluation of non-invasive atrial cardiomyopathy markers in relation to magnetic resonance imaging-defined cerebral stroke lesions.
- Among electrocardiographic, echocardiographic, and blood-based markers of atrial cardiomyopathy, amplified P-wave duration, P-wave area in lead II, PR interval, left atrial volume index, left atrial ejection fraction, and NT-proBNP were significantly associated with cerebral stroke lesions.
- When added to established clinical risk stratification including CHA_2_DS_2_-VA score, gender and history of prior atrial fibrillation non-invasive atrial cardiomyopathy markers provided marginal incremental discriminatory value, highlighting important limitations of their application in unselected populations with heterogeneous stroke mechanisms.

## Introduction

Atrial fibrillation (AF) is a common disease in older individuals and is associated with a significantly elevated risk of ischaemic stroke, which has a substantial impact on morbidity, mortality and quality of life.^1–3^ Strokes affect about 1.1 million people each year, and the mean global lifetime risk has increased to 24.9% over the past years.^4,5^ Approximately one-third of all ischaemic strokes are caused by AF, and as the Framingham study revealed, patients with AF have a three- to five-fold increased risk of stroke.^6,7^ Atrial fibrillation–related strokes are often more severe than non-AF-related strokes underlining the importance of adequate risk stratification.^6^ The clinical CHA_2_DS_2_-VA score, though widely used for stroke risk stratification in AF, has only moderate predictive accuracy.^8^ Moreover, increasing evidence suggests that atrial structural, functional, and electrophysiological abnormalities present in atrial cardiomyopathy (AtCM) may lead to atrial blood stasis and thromboembolism even in sinus rhythm.^9,10^ This hypothesis is supported by device-based studies that found no temporal association between AF episodes and acute ischaemic stroke, indicating that AtCM itself, rather than AF, may be the critical driver of embolic stroke risk.^11,12^ Consequently, both the latest European Society of Cardiology and American College of Cardiology guidelines for AF as well as two recently published AtCM consensus statements emphasize the importance of AtCM diagnosis for stroke risk assessment.^1–3,9,10^ The currently most established diagnostic methods for AtCM include invasive electroanatomical mapping and late gadolinium enhancement cardiac magnetic resonance imaging (MRI) both of which have been linked to increased rates of prior ischaemic strokes.^13,14^ However, these methods are time-consuming, expensive, and only suitable for certain patient groups. On the other hand, non-invasive, widely available, and cost-effective tools for AtCM diagnosis using electrocardiography (ECG), transthoracic echocardiography (TTE), or blood-based biomarkers have not been established as routine screening modalities for the general population due to limited clinical validation. Moreover, most population-based MRI cohorts comprise heterogeneous lesion mechanisms (small-vessel disease, large-artery atherosclerosis, and cardioembolism), and it remains unclear whether non-invasive AtCM surrogate markers retain incremental value beyond established vascular risk profiles in such real-world settings.

Therefore, we investigated (i) the association of non-invasive ECG-, TTE-, and blood biomarker-derived AtCM surrogate markers with cerebral stroke lesions on MRI and (ii) whether these markers improve discrimination beyond established clinical risk factors in a mixed-aetiology, population-based cohort of the Hamburg City Health Study (HCHS).

## Methods

### Study design and participants

**Figure 1:**
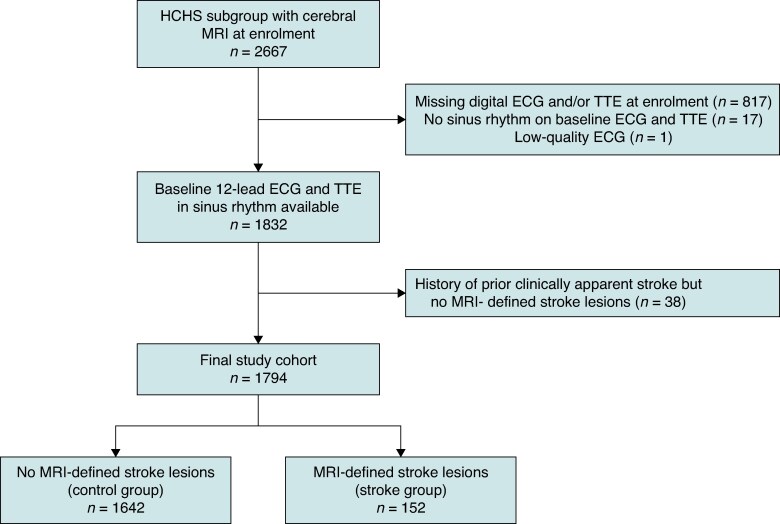
Study overview. Flowchart of participants included in the current study. ECG, electrocardiogram; HCHS, Hamburg City Health Study; TTE, transthoracic echocardiography; MRI, magnetic resonance imaging.

The HCHS is an ongoing large, prospective, long-term, population-based cohort study with the aim to obtain knowledge on important risk and prognostic factors in major chronic diseases.^15^ Since spring 2016, more than 45 000 individuals have been contacted via Hamburg’s residents’ registration office, and until now, 45 000 participants have been registered in the study. The HCHS includes a representative distribution across sex and age strata with participants aged 45–74 years and equal numbers of men and women. As part of the baseline examinations, a predefined subgroup with cardiovascular risk factors (European Society of Cardiology EuroSCORE > 4%) underwent standardized cerebral MRI for the assessment of stroke lesions at enrolment. To enable standardized ECG and TTE phenotyping of AtCM markers, we required availability of a digital 12-lead ECG and TTE performed at enrolment in sinus rhythm. Participants were excluded if digital ECG and/or TTE were missing at enrolment (*n* = 817), if sinus rhythm was not present on baseline ECG and TTE (*n* = 17), or if ECG quality did not allow reliable P-wave assessment (*n* = 1). This yielded 1832 participants with baseline 12-lead ECG and TTE in sinus rhythm available. Participants with self-reported history of clinically apparent stroke but without MRI-defined stroke lesions were excluded (*n* = 38) to reduce potential outcome misclassification in the control group. The final study cohort comprised 1794 participants. Within this cohort, individuals with MRI-defined stroke lesions constituted the stroke lesion group (*n* = 152), and those without MRI-defined stroke lesions constituted the control group (*n* = 1642). History of cardiovascular diagnoses was defined as documented diagnoses in medical records and/or participant self-report. An overview of the study population of our subgroup analysis is illustrated in *Figure 1*.

The study was approved by the local ethics committee of the Hamburg Chamber of Medical Practitioners (PV5131), and all participants provided written informed consent prior to study inclusion (identifier: NCT03934957 on clinicaltrials.gov).

### Magnetic resonance imaging diagnostics

A cerebral MRI was performed using a 3 Tesla Skyra MRI scanner (Siemens, Erlangen, Germany) as described previously.^16^ In brief, single-shell diffusion weighted imaging sequence was obtained using 75 axial slices covering the whole brain with gradients (*b* = 1000 s/mm^2^) applied along 64 non-collinear directions with the following parameters: repetition time = 8500 ms, echo time = 75 ms, slice thickness = 2 mm, in-plane resolution = 2 × 2 mm, and anterior-posterior phase-encoding direction. For 3D T1-weighted anatomical images, rapid acquisition gradient-echo sequence was used with the following sequence parameters: repetition time = 2500 ms, echo time = 2.12 ms, 256 axial slices, slice thickness = 0.94 mm, and in-plane resolution = 0.83 × 0.83 mm. 3D T2-weighted fluid attenuated inversion recovery images were measured with the following sequence parameters: repetition time = 4700 ms, echo time = 392 ms, 192 axial slices, slice thickness = 0.9 mm, and in-plane resolution = 0.75 × 0.75 mm.

The MRI findings were subsequently examined by two blinded neuroradiologists. Stroke lesions were defined as hyperintense lesions on T2-weighted fluid attenuated inversion recovery and a markedly hypointense signal intensity on T1-weighted images in a vascular distribution without mass effect or oedema.^17^ To better approximate potential stroke mechanisms, MRI-defined stroke lesions were further categorized according to lesion morphology and vascular imaging findings into likely cardioembolic (territorial infarcts or bilateral microembolic lesions), unlikely cardioembolic (lacunar or watershed infarcts, or any lesions associated with high-grade extra- or intracranial stenosis in the supplying vessel), and unclassified (ambiguous patterns).

### Non-invasive atrial cardiomyopathy diagnosis

Atrial cardiomyopathy was quantified using the currently most promising non-invasive tools in ECG, TTE, and blood biomarkers as stated in the current AtCM consensus documents.^9,10^

A standard digital 12-lead ECG (1 CS-200 Excellence, Schiller GmbH, Baar, Switzerland) in sinus rhythm was recorded. Conventional non-amplified P-wave duration (non-APWD), PR interval, P-wave terminal force in lead V1 (PTFV1), and P-wave area in lead II were analysed automatically by Schiller software (SEMA 3, Schiller GmbH, Baar, Switzerland). Amplified P-wave duration (APWD) and presence of advanced interatrial block were investigated manually by experts after digital ECG signal amplification to 80 mm/mV and a sweep speed of 175 mm/s using an automatically generated averaged beat, derived from all ECG single beats. Amplified P-wave duration was measured from the earliest initial deflection of any lead to latest end in any lead as described previously by our group.^18–21^ Subsequently, three AtCM stages depending on APWD were defined (<150 ms no relevant AtCM, 150–180 ms moderate AtCM, >180 ms severe AtCM).^18^ Advanced interatrial block was defined as occurrence of biphasic (positive-negative) P-waves in inferior leads II, III, and aVF.^22^

Transthoracic echocardiography (Acuson SC2000 Prime, Siemens, Erlangen, Germany) was performed in sinus rhythm in accordance with current guidelines from the European Association of Cardiovascular Imaging and the American Society of Echocardiography.^23^ Transthoracic echocardiography parameters were quantified by specially trained cardiologists and sonographers of the HCHS study group following standard operating procedures with regular internal quality controls.^24^ Left atrial and left ventricular ejection fraction were calculated using the disk summation technique based on biplane measurements (apical four- and two-chamber views).^23^ Left atrial global peak strain analysis was analysed using 2D speckle tracking.^25^

For blood-based biomarkers, high-sensitivity C-reactive protein (hsCRP) and N-terminal prohormone of brain natriuretic peptide (NT-proBNP) were measured from the initial blood sample.

All investigators were blinded to participant’s characteristics and clinical outcome.

### Objectives

The primary objective was to quantify the association of non-invasive ECG-, TTE-, and blood biomarker-derived AtCM surrogate markers with MRI-defined cerebral stroke lesions in participants with baseline sinus rhythm. The secondary objective was to evaluate whether these markers improve model discrimination beyond established clinical risk factors.

### Statistical analysis

Statistical analysis was performed using R v4.3.

Continuous variables are expressed as means ± standard deviation. Categorical variables are expressed with their absolute (in numbers) and relative frequency (in %). Student’s *t*-test and *χ*^2^ test were used to compare the two groups.

Univariable and multivariable logistic regression analyses were performed to identify both clinical parameters and AtCM markers significantly associated with stroke lesions on MRI. Multivariable regression analysis was performed including significant risk factors from clinical parameters, ECG, TTE, and blood-based biomarkers identified in univariable regression analyses. Results are reported as odds ratios and 95% confidence intervals (CIs). To assess the incremental discriminatory performance of the extended model (clinical risk factors plus non-invasive AtCM markers) compared with the clinical model alone, areas under the receiver operating characteristics curve (ROC-AUCs) were compared using DeLong’s test for correlated ROC curves. The difference in AUC (ΔAUC) was calculated with corresponding 95% CIs. To ensure comparability between models, these analyses were restricted to individuals with complete data available for all variables included in both the clinical and the extended model. In addition, decision curve analysis was performed to evaluate the net clinical benefit of both models across a range of clinically relevant threshold probabilities. Decision curve analysis was conducted using the same complete-case dataset as applied for the ΔAUC analysis.

All statistical analyses used a two-sided hypothesis and test significance was defined as *P* < 0.05.

## Results

A total of 1794 participants of the HCHS with cerebral MRI and corresponding digital ECG and TTE in sinus rhythm were included in the current study. Mean age of the total study cohort was 63.8 ± 8.4 years with the majority of participants being male (58.3%). Magnetic resonance imaging confirmed stroke lesions in a total of 152 (8.5%) participants who were included in the stroke group and compared to 1642 (91.5%) controls without stroke lesions in cerebral MRI.

### Clinical parameters and stroke risk

Compared to the control group, participants in the stroke group were significantly older (68.3 ± 6.4 vs. 63.4 ± 8.4 years, *P* < 0.001) and more often male (69.7% vs. 57.3%, *P* = 0.004). Additionally, a significant difference between the two groups was demonstrated in history of coronary artery disease (9.9% in the stroke group vs. 5.2% in controls, *P* = 0.022), prior myocardial infarction (7.9% vs. 3.5%, *P* = 0.013), and prior AF (10.5% vs. 3.6%, *P* < 0.001). Moreover, stenosis of cerebral vessels was significantly more present in the stroke group (10.5% vs. 2.1%, *P* < 0.001). Prior clinically apparent stroke was diagnosed in 19 (12.5%) participants of the stroke group. The remaining lesions represented clinically silent infarctions detected incidentally on MRI. The CHA_2_DS_2_-VA score was also statistically significantly different with 1.3 ± 0.9 in the stroke and 0.7 ± 0.8 in the control group (*P* < 0.001). Detailed clinical data of both the stroke and the control group are displayed in *Table 1*.

**Table 1:** Participant clinical characteristics

Variables	Total (*n* = 1794)	Control group (*n* = 1642)	Stroke group (*n* = 152)	*P* value
Age, years	63.8 ± 8.4	63.4 ± 8.4	68.3 ± 6.4	**<0**.**001**
Male sex, *n* (%)	1046 (58.3%)	940 (57.3%)	106 (69.7%)	**0**.**004**
Body mass index, kg/m^2^	26.8 ± 4.4	26.7 ± 4.3	27.4 ± 4.3	0.057
Smoking history, *n* (%)	1157 (64.5%)	1057 (64.4%)	100 (65.8%)	0.839
Coronary artery disease, *n* (%)	100 (5.6%)	85 (5.2%)	15 (9.9%)	**0**.**022**
History of myocardial infarction, *n* (%)	69 (3.9%)	57 (3.5%)	12 (7.9%)	**0**.**013**
History of atrial fibrillation, *n* (%)	75 (4.2%)	59 (3.6%)	16 (10.5%)	**<0**.**001**
Heart failure, *n* (%)	54 (3.0%)	50 (3.1%)	4 (2.6%)	0.970
Stenosis of cerebral vessels, *n* (%)	50 (2.8%)	34 (2.1%)	16 (10.5%)	**<0**.**001**
History of clinically apparent stroke, *n* (%)	19 (1.1%)	0 (0%)	19 (12.5%)	**<0**.**001**
CHA_2_DS_2_-VA score	0.8 ± 0.8	0.7 ± 0.8	1.3 ± 0.9	**<0**.**001**
Left ventricular ejection fraction, %	59 ± 5	59 ± 5	58 ± 5	0.770

### Electrocardiography-based atrial cardiomyopathy markers and stroke risk

**Figure 2:**
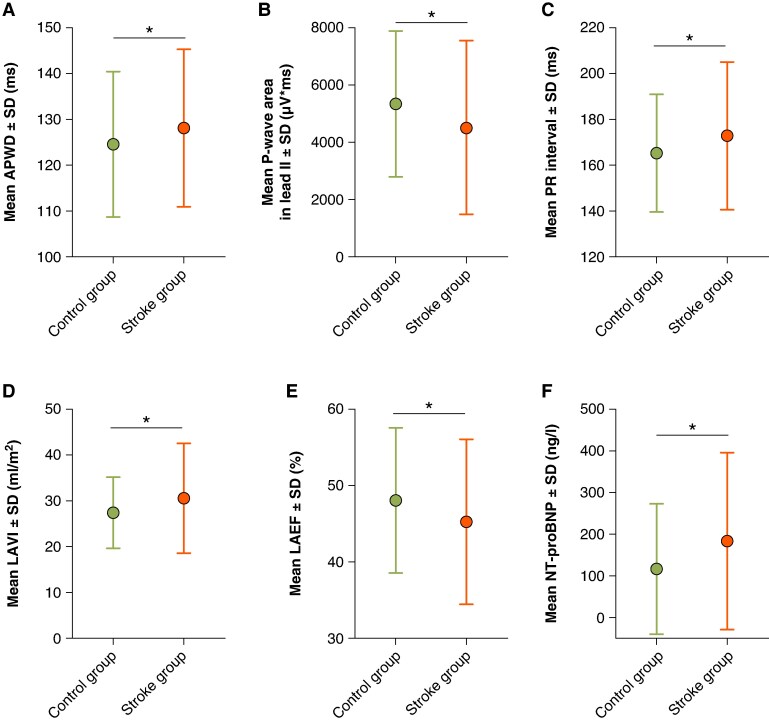
Non-invasive AtCM markers in participants with and without cerebral stroke lesions. Comparison of non-invasive markers of AtCM between participants of control group (in green) and stroke group (in red). Bar plots show the group-wise means ± standard deviation (SD) for APWD (*A*), P-wave area in lead II (*B*), PR interval (*C*), left atrial volume index (*D*), left atrial ejection fraction (*E*), and NT-proBNP (*F*). Participants with stroke lesions (stroke group) showed significantly different values across all six AtCM markers compared to the control group (*P < 0.05 for all comparisons).

Amplified P-wave duration was significantly different in both groups with a length of 128 ± 17 ms in the stroke group and 125 ± 16 ms in controls (*P* = 0.015; *Figure 2A*). Participants in the stroke group were slightly more frequently classified into higher AtCM stages based on APWD without reaching statistical significance (*P* = 0.065). There was no relevant difference in the appearance of advanced interatrial block (3.9% vs. 2.6%, *P* = 0.451).

Conventional non-APWD was not statistically significant between both groups (117 ± 24 ms vs. 116 ± 17 ms, *P* = 0.570) underlining the importance of amplification. Moreover, P-wave area in lead II was significantly lower in participants of the stroke group compared to controls (4516 ± 3034 vs. 5339 ± 2541 µV·ms, *P* = 0.002; *Figure 2B*). PR interval was significantly longer in the stroke group (173 ± 32 ms vs. 165 ± 26 ms, *P* < 0.001; *Figure 2C*). All analysed ECG markers for AtCM are listed in *Table 2*.

**Table 2:** Non-invasive AtCM parameters

Variables	Total (*n* = 1794)	Control group (*n* = 1642)	Stroke group (*n* = 152)	*P* value
ECG-based parameters				
Manual amplified *P*-wave analysis				
APWD, ms	125 ± 16	125 ± 16	128 ± 17	**0**.**015**
Advanced interatrial block, *n* (%)	48 (2.7%)	42 (2.6%)	6 (3.9%)	0.451
AtCM stages, *n* (%)				0.065
AtCM stage I (APWD < 150 ms)	1688 (94.1%)	1551 (94.5%)	137 (90.1%)	
AtCM stage II (APWD 150–180 ms)	91 (5.1%)	79 (4.8%)	12 (7.9%)	
AtCM stage III (APWD > 180 ms)	15 (0.8%)	12 (0.7%)	3 (2.0%)	
Automatic ECG-analysis				
Non-APWD, ms	116 ± 18	116 ± 17	117 ± 24	0.570
P-wave terminal force in V1, µV·ms	2376 ± 2050	2347 ± 2038	2686 ± 2166	0.073
P-wave area in lead II, µV·ms	5268 ± 2596	5339 ± 2541	4516 ± 3034	**0**.**002**
PR interval, ms	166 ± 26	165 ± 26	173 ± 32	**<0**.**001**
TTE-based parameters				
Left atrial volume index, mL/m^2^	28 ± 8	28 ± 8	31 ± 12	**0**.**038**
Left atrial ejection fraction, %	48 ± 10	48 ± 10	45 ± 11	**0**.**032**
Left atrial global peak strain, %	39.9 ± 14.4	40.0 ± 14.6	37.8 ± 12.5	0.168
Blood-based biomarkers				
hsCRP, mg/dL	0.24 ± 0.44	0.23 ± 0.40	0.31 ± 0.75	0.220
NT-proBNP, ng/L	122.9 ± 162.8	117.1 ± 156.2	183.7 ± 212.6	**<0**.**001**

APWD, amplified P-wave duration; AtCM, atrial cardiomyopathy; hsCRP, high-sensitivity C-reactive protein; NT-proBNP, N-terminal prohormone of brain natriuretic peptide. Bold values indicate statistical significance.

In univariable regression analysis including all ECG parameters, APWD, P-wave area in lead II, and PR interval were statistically significant risk factors of stroke lesions (*Table 3*).

**Table 3:** Univariable regression analyses of significant clinical parameters and AtCM markers for stroke lesions

Variable	Univariable regression analyses		
	OR	95% CI	*P* value
Age per year	1.08	1.06–1.11	**<0**.**001**
Male sex	1.72	1.20–2.44	**0**.**003**
History of prior atrial fibrillation	3.17	1.72–5.53	**<0**.**001**
CHA_2_DS_2_-VA score per point	2.00	1.67–2.40	**<0**.**001**
APWD per 10 ms	1.13	1.03–1.24	**0**.**009**
P-wave area in lead II per 100 µV·ms	0.99	0.98–1.00	**<0**.**001**
PR interval per 10 ms	1.10	1.01–1.22	**<0**.**001**
Left atrial volume index per mL/m^2^	1.04	1.02–1.06	**<0**.**001**
Left atrial ejection fraction per %	0.97	0.95–0.99	**0**.**010**
NT-proBNP per 10 ng/L	1.02	1.01–1.02	**<0**.**001**

APWD, amplified P-wave duration; NT-pro-BNP, N-terminal prohormone of brain natriuretic peptide; OR, odds ratio; CI, confidence interval. Bold values indicate statistical significance.

### Transthoracic echocardiography–based atrial cardiomyopathy markers and stroke risk

Left atrial volume index was significantly higher in the stroke cohort compared to controls (31 ± 12 mL/m^2^ vs. 28 ± 8 mL/m^2^, *P* = 0.038; *Figure 2D* and *Table 2*), and left atrial ejection fraction was significantly lower in the stroke group (45 ± 11% vs. 48 ± 10%, *P* = 0.032, *Figure 2E* and *Table 2*). Left atrial global peak strain however was not significantly different between the two groups (37.8 ± 12.5% vs. 40.0 ± 14.6%, *P* = 0.168; *Table 2*).

In univariable regression analysis, left atrial volume index and left atrial ejection fraction were statistically significant risk factors for stroke lesions (*Table 3*).

### Blood-based atrial cardiomyopathy biomarkers and stroke risk

Considering the blood-based parameters, NT-proBNP was significantly different between the stroke group and controls (183.7 ± 212.6 ng/L in the stroke group vs. 117.1 ± 156.2 ng/L in controls, *P* < 0.001; *Figure 2F* and *Table 2*) whereas hsCRP was not significantly different (0.31 ± 0.75 mg/dL vs. 0.23 ± 0.40 mg/dL, *P* = 0.220; *Table 2*). In univariable regression analysis, NT-proBNP was also a significant risk factor for stroke lesions (*Table 3*).

### Best risk prediction model for stroke including clinical parameters and non-invasive atrial cardiomyopathy markers

**Figure 3:**
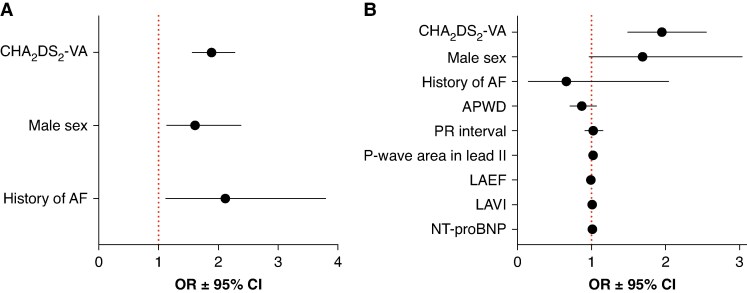
Association of clinical risk factors and non-invasive AtCM markers with cerebral stroke lesions. Forest plots display ORs and 95% CIs from two logistic regression models including statistically significant clinical risk factors as well as significant AtCM markers from ECG-, TTE-, and blood-based biomarkers (*A*: clinical model including CHA_2_DS_2_-VA score, male sex, and history of prior AF); *B*: extended model including CHA_2_DS_2_-VA score, male sex, history of prior AF, APWD, PR interval, P-wave area in lead II, left atrial ejection fraction, left atrial volume index, and NT-proBNP. All markers were standardized prior to analysis.

In univariable regression analysis, several clinical parameters were significantly associated with the presence of stroke lesions on cerebral MRI (*Table 3*). To avoid collinearity, only the composite CHA_2_DS_2_-VA score, rather than its individual components, was included in the multivariable clinical model in addition to history of prior AF and male sex (*Table 4*and *Figure 3A*).

**Table 4:** Multivariable regression analysis including significant clinical parameters for stroke lesions

	Multivariable regression analysis		
Variable	OR	95% CI	*P* value
CHA_2_DS_2_-VA score per point	1.89	1.57–2.27	**<0**.**001**
History of prior atrial fibrillation	2.12	1.12–3.80	**0**.**014**
Male sex	1.61	1.14–2.38	**0**.**010**

OR, odds ratio; CI, confidence interval. Bold values indicate statistical significance.

When significant risk factors identified from ECG-, TTE-, and blood-based AtCM markers in the univariable analyses were added to the extended model, only the CHA_2_DS_2_-VA score and P-wave area in lead II remained independently associated with stroke lesions (*Table 5* and *Figure 3B*). A total of 970 participants had complete data available for all variables included in both models and were therefore analysed in the model comparison. In this complete-case dataset, the ROC-AUC of the clinical parameter model was 0.680. Addition of non-invasive AtCM markers increased the ROC-AUC of the extended model to 0.710 (ΔAUC 0.030, 95% CI: 0.0001–0.0594, *P* = 0.049). Although statistically significant, the absolute improvement in discrimination was modest. Decision curve analysis also did not demonstrate a consistent or clinically meaningful improvement in net benefit of the extended model compared with the clinical model (see Supplementary material online, *Figure S1*).

**Table 5:** Multivariable regression analysis including significant clinical parameters and AtCM markers for stroke lesions

	Multivariable regression analysis		
Variable	OR	95% CI	*P* value
CHA_2_DS_2_-VA score per point	1.95	1.49–2.55	**<0**.**001**
History of prior atrial fibrillation	0.66	0.15–2.04	0.524
Male sex	1.69	0.97–3.03	0.067
APWD per 10 ms	0.90	0.82–1.11	0.486
P-wave area in lead II per 100 µV·ms	0.99	0.98–1.00	**0**.**031**
PR interval per 10 ms	1.01	0.92–1.11	0.827
Left atrial volume index per mL/m^2^	1.02	0.99–1.05	0.257
Left atrial ejection fraction per %	0.99	0.97–1.02	0.485
NT-proBNP per 10 ng/L	1.00	1.00–1.01	0.420

APWD, amplified P-wave duration; NT-pro-BNP, N-terminal prohormone of brain natriuretic peptide; OR, odds ratio; CI, confidence interval. Bold values indicate statistical significance.

To evaluate the association of AtCM surrogate markers with MRI-defined stroke lesions independent of diagnosed AF, we additionally performed a sensitivity analysis excluding all participants with history of prior AF. This AF-excluded cohort comprised 1719 participants, of whom 136 (7.9%) had MRI-defined stroke lesions and 1583 (92.1%) served as controls and yielded findings consistent with those observed in the cohort including participants with history of prior AF (see Supplementary material online, *Table S1*). In univariable analyses, age, CHA_2_DS_2_-VA score, male sex, APWD, P-wave area in lead II, PR interval, left atrial volume index, left atrial ejection fraction, and NT-proBNP remained significantly associated with MRI-defined stroke lesions (see Supplementary material online, *Table S1a*). In the multivariable clinical model, CHA_2_DS_2_-VA score and male sex remained independent predictors (see Supplementary material online, *Table S1b*). After adding AtCM surrogate markers (APWD, P-wave area in lead II, PR interval, left atrial volume index, left atrial ejection fraction, and NT-proBNP), only CHA_2_DS_2_-VA score and P-wave area in lead II remained independently associated with stroke lesions (see Supplementary material online, *Table S1*c).

### Stroke lesion pattern subgroup and sex-stratified analyses

Among participants with MRI-defined stroke lesions (*n* = 152), 37 were classified as likely cardioembolic, 84 as unlikely cardioembolic, and 31 as unclassified.

In a two-group comparison between likely and unlikely cardioembolic lesions, neither clinical risk factors nor ECG-, TTE-, or blood-based AtCM surrogate markers differed significantly between the groups (see Supplementary material online, *Table S2*).

Significant sex-related differences were observed for several AtCM parameters including amplified and non-APWD, P-wave terminal force in V1, P-wave area in lead II, PR interval, left atrial volume index, left atrial global peak strain, and NT-proBNP (see Supplementary material online, *Table S3*). Using ROC-AUC analysis with the Youden index, exploratory sex-specific optimal cut-off values were determined for the endpoint of MRI-defined stroke lesions (see Supplementary material online, *Table S3*).

## Discussion

This analysis was designed to test the incremental value of widely available, non-invasive AtCM surrogate markers for the detection of MRI-defined stroke lesions in a population-based cohort with heterogeneous lesion mechanisms. In this real-world setting, established clinical risk burden, as reflected by the CHA_2_DS_2_-VA score, male sex, and history of prior AF accounted for most of the discriminatory performance, whereas AtCM surrogate markers provided only marginal incremental value.

Specifically, we report three main findings:

1. Stroke lesions detected by cerebral MRI were present in almost 10% of participants (mean age 63.8 years) within a subgroup of the prospective, population-based HCHS.
2. Higher CHA_2_DS_2_-VA score, history of prior AF, and male sex, as established clinical risk factors, were independently associated with the presence of stroke lesions.
3. Among non-invasive AtCM markers, only P-wave area in lead II remained an independent predictor when added to the clinical risk factor model, resulting in only a marginal improvement in discrimination for identifying individuals with stroke lesions (ΔAUC: 0.03, 95% CI: 0.0001–0.0594).

### Clinical relevance of stroke

Stroke is one of the leading causes of death in the European Union and the USA and a leading cause of disability in adults.^4,5^ Considering the growing and aging population, stroke events and their long-term consequences, including health care costs, are expected to increase exceptionally.^4^ In the current study, cerebral stroke lesions were identified on MRI in 8.5% of participants enrolled in the prospective epidemiological HCHS, which is in line with previous population-based studies reporting a prevalence of stroke lesions of 10–20%.^26^ Among these individuals, 12.5% had a documented history of clinically apparent stroke underlining the clinical relevance. The remaining 87.5% of individuals in the stroke group had clinically silent cerebral lesions that were incidentally detected on brain MRI. Despite the absence of acute neurological symptoms, silent infarctions represent clinically relevant cerebrovascular injury and have been consistently associated with adverse outcomes, including increased risk of future symptomatic stroke and mortality, cognitive decline, and accelerated neurobehavioral disturbances.^27^

### Risk factors for stroke

In the present study, established clinical risk factors including CHA_2_DS_2_-VA score, male sex, and history of prior AF were confirmed as strong predictors of stroke lesions on cerebral MRI. These findings are consistent with the well-documented role of AF and vascular comorbidities in the pathogenesis of ischaemic stroke.^28^

The concept of AtCM has gained attention in recent years as a potential contributor to stroke risk, independent of AF.^9,10,29^ In our study, we evaluated multiple electrical, structural, functional, and blood-based biomarker-derived parameters as surrogates of AtCM, but only the ECG-derived parameter P-wave area in lead II was significantly associated with stroke lesions in addition to the CHA_2_DS_2_-VA score after multivariable adjustment. This finding suggests that electrical remodelling may capture aspects of AtCM relevant to stroke lesions more robustly than structural or biochemical surrogates in this setting and was consistent after exclusion of participants with a prior AF diagnosis, suggesting that the observed findings are not merely driven by known AF. According to the recent European Heart Rhythm Association consensus framework, AtCM can be conceptualized in stages.^9^ In our cohort, predominantly comprising individuals without known AF, most detectable alterations were confined to electrical parameters, consistent with a mild or subclinical stage of AtCM. The inverse association between P-wave area in lead II and stroke lesions likely reflects reduced atrial electrical mass secondary to fibrotic remodelling, which represents a hallmark of AtCM. These findings align with prior studies linking P-wave morphology to atrial fibrosis and thromboembolic risk.^9,10^ In contrast, more advanced structural or functional markers such as left atrial volume index, left atrial ejection fraction, left atrial strain, and blood-based biomarkers such as NT-proBNP did not remain independently associated after multivariable adjustment, suggesting that in a population-based setting without overt atrial disease, early electrical changes may precede clinically manifest structural remodelling. However, when added to a model containing only clinical risk factors, the non-invasive AtCM markers increased the model’s diagnostic performance only marginally (ΔAUC of 0.03). It should be acknowledged that this model comparison was restricted to 970 of the 1794 participants with complete data available for all variables included in both models. While this approach ensured comparability between both models, it substantially reduced the effective sample size. Moreover, the requirement for complete multimodal phenotyping highlights a potential challenge for real-world implementation of comprehensive AtCM assessment in population-based settings, where availability of all modalities may be limited. Nevertheless, the modest gain after inclusion of AtCM markers in the clinical risk factor model suggests that, in a heterogeneous cohort with multiple stroke mechanisms, the clinical relevance of AtCM might be diluted by other prevalent risk factors such as large-artery atherosclerosis and small-vessel disease. In our study, individuals in the stroke group more frequently had coronary artery disease, prior myocardial infarction, and cerebral vessel stenosis, all of which are known contributors to atherosclerotic brain injury.^28^ Given that only 10.5% of stroke patients had documented history of prior AF and the mean CHA_2_DS_2_-VA score was relatively low, many lesions likely arose from non-cardioembolic causes. This heterogeneity may explain why AtCM markers, although significant, were not strong discriminators in our population demonstrating important limitations for the clinical translation of AtCM surrogate markers in unselected populations. These findings complement recently published large-scale outcome studies investigating AtCM in AF cohorts. In the EAST-AFNET 4 trial, AtCM severity (defined by left atrial enlargement, prolonged PR interval, and elevated NT-proBNP) was associated with adverse cardiovascular outcomes, including stroke, in patients with recently diagnosed AF.^29^ Relander *et al*. demonstrated that various P-wave abnormalities were able to predict future stroke in patients with AF undergoing electrical cardioversion.^30^ In such populations, electrical, structural, and blood-biomarker-based AtCM surrogates likely play a more prominent role, as manifest atrial pathology is already present and the probability of a cardioembolic stroke mechanism is consequently higher compared with our predominantly community-based cohort without known AF. However, even after stroke lesion pattern stratification aiming to enrich for likely cardioembolic infarcts, AtCM surrogate markers did not demonstrate stronger separation between mechanistic subgroups in our study. It must be noted that these subgroup analyses were limited by small sample sizes and should be interpreted with caution. In addition, lesion morphology alone does not allow definitive determination of stroke aetiology, particularly in a population-based cohort largely consisting of individuals without previously diagnosed AF and without formal aetiological adjudication. Nevertheless, our findings may provide a possible explanation for the negative results of the ARCADIA trial, in which anticoagulation did not reduce recurrent stroke compared with aspirin in patients with evidence for AtCM.^31^

Another important finding of our study was that gender impacts multiple non-invasive AtCM parameters. We could demonstrate that not only structural remodelling parameters are influenced but also electrical, functional, and blood-based biomarkers of AtCM. Currently available consensus documents emphasize that no universally established diagnostic cut-offs for AtCM exist.^9,10^ Our findings further highlight that uniform thresholds may be biologically inappropriate. The proposed sex-specific thresholds should be considered exploratory and hypothesis-generating, as external validation in independent cohorts is required before incorporation into risk stratification algorithms.

Moreover, advanced artificial intelligence–based multimodal phenotyping approaches may further improve risk stratification in large imaging cohorts. Future studies integrating explainable artificial intelligence techniques with mechanistically grounded AtCM markers may provide complementary insights.

### Limitations

First, this is an observational cross-sectional analysis. Therefore, causality cannot be inferred. Second, although the participants included in the present study exhibited cardiovascular risk factors, they nevertheless represented a relatively young and healthy sample from a prospective population-based cohort. Consequently, the proportion of individuals with stroke lesions and the generalizability of these findings to a potentially older and more severely ill population are limited. Third, while we performed a subgroup analysis based on stroke lesion morphology and vascular imaging findings to approximate potential cardioembolic mechanisms, definitive aetiologic adjudication was not available. Therefore, the true aetiology of the diagnosed strokes remains uncertain in the analysis, precluding a definitive assessment of whether they were cardioembolic in origin and thus potentially attributable to an underlying AtCM, particularly in a general population-based cohort largely comprising individuals without previously diagnosed AF. Fourth, restricting ECG and TTE phenotyping to participants in sinus rhythm at enrolment enables standardized AtCM marker measurements but preferentially captures participants with paroxysmal (rather than persistent) AF among those with a history of prior AF. However, this approach was necessary to allow mechanistic assessment of AtCM and likely biases the results towards underestimation rather than overestimation of diagnostic performance. Additionally, according to the study design, it was not possible to assess AF burden, a metric of growing interest beyond the dichotomous analysis based on presence/absence of AF.^32^

## Conclusion

In this population-based cohort, multiple non-invasive AtCM surrogate markers were associated with stroke lesions on MRI in univariable analyses. However, beyond established clinical risk factors, their incremental diagnostic value was modest. These findings highlight limitations of current non-invasive AtCM surrogate markers for stroke lesion detection in unselected populations with heterogeneous lesion mechanisms.

## Supplementary Material

euag063_Supplementary_Data

## Data Availability

The data underlying this article will be shared on reasonable request to the corresponding authors.

## References

[1] Van Gelder IC, Rienstra M, Bunting KV, Casado-Arroyo R, Caso V, Crijns H et al 2024 ESC guidelines for the management of atrial fibrillation developed in collaboration with the European association for cardio-thoracic surgery (EACTS). Eur Heart J 2024;45:3314–414. 39210723 10.1093/eurheartj/ehae176

[2] Rienstra M, Tzeis S, Bunting KV, Caso V, Crijns H, De Potter TJR et al Spotlight on the 2024 ESC/EACTS management of atrial fibrillation guidelines: 10 novel key aspects. Europace 2024;26:euae298. 39716733 10.1093/europace/euae298 PMC11666470

[3] Joglar JA, Chung MK, Armbruster AL, Benjamin EJ, Chyou JY, Cronin EM et al 2023 ACC/AHA/ACCP/HRS guideline for the diagnosis and management of atrial fibrillation: a report of the American College of Cardiology/American Heart Association joint committee on clinical practice guidelines. Circulation 2024;149:e1–156. 38033089 10.1161/CIR.0000000000001193 PMC11095842

[4] Wafa HA, Wolfe CDA, Emmett E, Roth GA, Johnson CO, Wang Y. Burden of stroke in Europe: thirty-year projections of incidence, prevalence, deaths, and disability-adjusted life years. Stroke 2020;51:2418–27. 32646325 10.1161/STROKEAHA.120.029606 PMC7382540

[5] Virani SS, Alonso A, Benjamin EJ, Bittencourt MS, Callaway CW, Carson AP et al Heart disease and stroke statistics-2020 update: a report from the American Heart Association. Circulation 2020;141:e139–596. 31992061 10.1161/CIR.0000000000000757

[6] Hahne K, Monnig G, Samol A. Atrial fibrillation and silent stroke: links, risks, and challenges. Vasc Health Risk Manag 2016;12:65–74. 27022272 10.2147/VHRM.S81807 PMC4788372

[7] Wolf PA, Abbott RD, Kannel WB. Atrial fibrillation as an independent risk factor for stroke: the framingham study. Stroke 1991;22:983–8. 1866765 10.1161/01.str.22.8.983

[8] Lip GY, Nieuwlaat R, Pisters R, Lane DA, Crijns HJ. Refining clinical risk stratification for predicting stroke and thromboembolism in atrial fibrillation using a novel risk factor-based approach: the euro heart survey on atrial fibrillation. Chest 2010;137:263–72. 19762550 10.1378/chest.09-1584

[9] Goette A, Corradi D, Dobrev D, Aguinaga L, Cabrera JA, Chugh SS et al Atrial cardiomyopathy revisited-evolution of a concept: a clinical consensus statement of the European heart rhythm association (EHRA) of the ESC, the heart rhythm society (HRS), the Asian Pacific heart rhythm society (APHRS), and the Latin American heart rhythm society (LAHRS). Europace 2024;26:euae204. 39077825 10.1093/europace/euae204 PMC11431804

[10] Weerts J, Tica O, Aranyo J, Basile C, Borizanova-Petkova A, Borovac JA et al Atrial cardiomyopathy: from healthy atria to atrial failure. A clinical consensus statement of the heart failure association of the ESC. Eur J Heart Fail 2025;27:2173–94. 40763073 10.1002/ejhf.3782 PMC12765236

[11] Martin DT, Bersohn MM, Waldo AL, Wathen MS, Choucair WK, Lip GY et al Randomized trial of atrial arrhythmia monitoring to guide anticoagulation in patients with implanted defibrillator and cardiac resynchronization devices. Eur Heart J 2015;36:1660–8. 25908774 10.1093/eurheartj/ehv115

[12] Brambatti M, Connolly SJ, Gold MR, Morillo CA, Capucci A, Muto C et al Temporal relationship between subclinical atrial fibrillation and embolic events. Circulation 2014;129:2094–9. 24633881 10.1161/CIRCULATIONAHA.113.007825

[13] King JB, Azadani PN, Suksaranjit P, Bress AP, Witt DM, Han FT et al Left atrial fibrosis and risk of cerebrovascular and cardiovascular events in patients with atrial fibrillation. J Am Coll Cardiol 2017;70:1311–21. 28882227 10.1016/j.jacc.2017.07.758

[14] Muller P, Makimoto H, Dietrich JW, Fochler F, Nentwich K, Krug J et al Association of left atrial low-voltage area and thromboembolic risk in patients with atrial fibrillation. Europace 2018;20:f359–65. 29016757 10.1093/europace/eux172

[15] Jagodzinski A, Johansen C, Koch-Gromus U, Aarabi G, Adam G, Anders S et al Rationale and design of the Hamburg city health study. Eur J Epidemiol 2020;35:169–81. 31705407 10.1007/s10654-019-00577-4 PMC7125064

[16] Petersen M, Frey BM, Schlemm E, Mayer C, Hanning U, Engelke K et al Network localisation of white matter damage in cerebral small vessel disease. Sci Rep 2020;10:9210. 32514044 10.1038/s41598-020-66013-w PMC7280237

[17] Allen LM, Hasso AN, Handwerker J, Farid H. Sequence-specific MR imaging findings that are useful in dating ischemic stroke. Radiographics 2012;32:1285–97. discussion 1297–1289. 22977018 10.1148/rg.325115760

[18] Muller-Edenborn B, Minners J, Keyl C, Eichenlaub M, Jander N, Abdelrazek S et al Electrocardiographic diagnosis of atrial cardiomyopathy to predict atrial contractile dysfunction, thrombogenesis and adverse cardiovascular outcomes. Sci Rep 2022;12:576. 35022443 10.1038/s41598-021-04535-7 PMC8755780

[19] Eichenlaub M, Mueller-Edenborn B, Lehrmann H, Minners J, Nairn D, Loewe A et al Non-invasive body surface electrocardiographic imaging for diagnosis of atrial cardiomyopathy. Europace 2021;23:2010–9. 34463710 10.1093/europace/euab140

[20] Eichenlaub M, Mueller-Edenborn B, Minners J, Jander N, Allgeier M, Lehrmann H et al Left atrial hypertension, electrical conduction slowing, and mechanical dysfunction—the pathophysiological triad in atrial fibrillation-associated atrial cardiomyopathy. Front Physiol 2021;12:670527. 34421634 10.3389/fphys.2021.670527 PMC8375593

[21] Jadidi A, Muller-Edenborn B, Chen J, Keyl C, Weber R, Allgeier J et al The duration of the amplified Sinus-P-wave identifies presence of left atrial low voltage substrate and predicts outcome after pulmonary vein isolation in patients with persistent atrial fibrillation. JACC Clin Electrophysiol 2018;4:531–43. 30067494 10.1016/j.jacep.2017.12.001

[22] Bayes de Luna A, Platonov P, Cosio FG, Cygankiewicz I, Pastore C, Baranowski R et al Interatrial blocks. A separate entity from left atrial enlargement: a consensus report. J Electrocardiol 2012;45:445–51. 22920783 10.1016/j.jelectrocard.2012.06.029

[23] Lang RM, Badano LP, Mor-Avi V, Afilalo J, Armstrong A, Ernande L et al Recommendations for cardiac chamber quantification by echocardiography in adults: an update from the American society of echocardiography and the European association of cardiovascular imaging. J Am Soc Echocardiogr 2015;28:1–39.e14. 25559473 10.1016/j.echo.2014.10.003

[24] Ohlrogge AH, Camen S, Nagel L, Brederecke J, Jensen M, Stenmans E et al Subtle signs of atrial cardiomyopathy and left ventricular diastolic dysfunction are associated with reduced cognitive function: results from the Hamburg city health study. Clin Res Cardiol 2025;114:1658–70. 39601872 10.1007/s00392-024-02581-5 PMC12708828

[25] Badano LP, Kolias TJ, Muraru D, Abraham TP, Aurigemma G, Edvardsen T et al Standardization of left atrial, right ventricular, and right atrial deformation imaging using two-dimensional speckle tracking echocardiography: a consensus document of the EACVI/ASE/industry task force to standardize deformation imaging. Eur Heart J Cardiovasc Imaging 2018;19:591–600. 29596561 10.1093/ehjci/jey042

[26] Fanning JP, Wong AA, Fraser JF. The epidemiology of silent brain infarction: a systematic review of population-based cohorts. BMC Med 2014;12:119. 25012298 10.1186/s12916-014-0119-0 PMC4226994

[27] Huang J, Biessels GJ, de Leeuw FE, Ii Y, Skoog I, Mok V et al Cerebral microinfarcts revisited: detection, causes, and clinical relevance. Int J Stroke 2024;19:7–15. 37470314 10.1177/17474930231187979

[28] O'Donnell MJ, Xavier D, Liu L, Zhang H, Chin SL, Rao-Melacini P et al Risk factors for ischaemic and intracerebral haemorrhagic stroke in 22 countries (the INTERSTROKE study): a case-control study. Lancet 2010;376:112–23. 20561675 10.1016/S0140-6736(10)60834-3

[29] Goette A, Lemoine MD, Borof K, Schotten U, Breithardt G, Camm AJ et al Prevalence and severity of atrial cardiomyopathy in patients with recently diagnosed atrial fibrillation and stroke risk factors and its association with early rhythm control: a secondary analysis of EAST-AFNET 4. Europace 2025;27:euaf256. 41061672 10.1093/europace/euaf256 PMC12559886

[30] Relander A, Ruohonen I, Jaakkola S, Vasankari T, Nuotio I, Airaksinen KEJ et al Novel electrocardiographic classification for stroke prediction in atrial fibrillation patients undergoing cardioversion. Heart Rhythm 2024;21:2407–18. 38677357 10.1016/j.hrthm.2024.04.083

[31] Kamel H, Longstreth WT Jr., Tirschwell DL, Kronmal RA, Marshall RS, Broderick JP et al Apixaban to prevent recurrence after cryptogenic stroke in patients with atrial cardiopathy: the ARCADIA randomized clinical trial. JAMA 2024;331:573–81. 38324415 10.1001/jama.2023.27188 PMC10851142

[32] Doehner W, Boriani G, Potpara T, Blomstrom-Lundqvist C, Passman R, Sposato LA et al Atrial fibrillation burden in clinical practice, research, and technology development: a clinical consensus statement of the European Society of Cardiology council on stroke and the European heart rhythm association. Europace 2025;27:euaf019. 40073206 10.1093/europace/euaf019 PMC11901050

