# Supplementary material for: Association of non-invasive atrial cardiomyopathy markers with cerebral stroke lesions: a population-based analysis from the Hamburg City Health Study
Source: Europace. 2026 Mar 27;28(4):euag063. doi: 10.1093/europace/euag063 (PMC13122356; doi:10.1093/europace/euag063)
Supplement: euag063_Supplementary_Data [file euag063_supplementary_data.docx]

**tabSupplementary Data**

**Table S1. Uni- and multivariable regression analyses after exclusion of participants with history of prior atrial fibrillation.**

**Table S1a.** Univariable regression analyses of significant clinical parameters and atrial cardiomyopathy markers for stroke lesions.

| **Variable** | **Univariable regression analyses** | | |
| --- | --- | --- | --- |
|  | **OR** | **95% CI** | **P value** |
| Age per year | 1.08 | 1.05-1.11 | **<0.001** |
| Male sex | 1.72 | 1.19-2.56 | **0.004** |
| CHA_2_DS_2_-VA score per point | 2.05 | 1.70-2.49 | **<0.001** |
| APWD per 10 ms | 1.12 | 1.01-1.24 | **0.028** |
| P-wave area in lead II per 100 µV·ms | 0.99 | 0.98-1.00 | **<0.001** |
| PR interval per 10 ms | 1.11 | 1.10-1.22 | **<0.001** |
| Left atrial volume index per ml/m^2^ | 1.03 | 1.00-1.05 | **0.050** |
| Left atrial ejection fraction per % | 0.98 | 0.95-1.00 | **0.038** |
| NT-proBNP per 10 ng/l | 1.01 | 1.00-1.02 | **0.004** |

APWD amplified P-wave duration, NT-pro-BNP N-terminal prohormone of brain natriuretic peptide, OR Odds Ratio, CI Confidence Interval

**Table S1b**. Multivariable regression analysis including significant clinical parameters for stroke lesions.

|  | **Multivariable regression analysis** | |  |  |
| --- | --- | --- | --- | --- |
| **Variable** | **OR** | **95% CI** | | **P Value** |
| CHA_2_DS_2_-VA score per point | 2.02 | 1.67-2.45 | | **<0.001** |
| Male sex | 1.61 | 1.10-2.38 | | **0.017** |

OR Odds Ratio, CI Confidence Interval

**Table S1c.** Multivariable regression analysis including significant clinical parameters and atrial cardiomyopathy markers for stroke lesions.

|  | **Multivariable regression analysis** | | |
| --- | --- | --- | --- |
| **Variable** | **OR** | **95% CI** | **P Value** |
| CHA_2_DS_2_-VA score per point | 1.98 | 1.50-2.60 | **<0.001** |
| Male sex | 1.72 | 0.98-3.13 | 0.065 |
| APWD per 10 ms | 0.94 | 0.79-1.12 | 0.481 |
| P-wave area in lead II per 100 µV·ms | 0.99 | 0.98-1.00 | **0.033** |
| PR interval per 10 ms | 1.02 | 0.92-1.12 | 0.752 |
| Left atrial volume index per ml/m^2^ | 1.01 | 0.98-1.04 | 0.482 |
| Left atrial ejection fraction per % | 0.99 | 0.96-1.01 | 0.341 |
| NT-proBNP per 10 ng/l | 1.01 | 0.99-1.02 | 0.344 |

APWD amplified P-wave duration, NT-pro-BNP N-terminal prohormone of brain natriuretic peptide, OR odds ratio, CI confidence interval

**Table S2. Baseline clinical characteristics and atrial cardiomyopathy parameters of participants with magnetic resonance imaging-defined stroke lesions stratified by lesion pattern.**

| **Variables** | **Overall**  **(N=152)** | **Unclassified (N=31)** | **Likely cardioembolic**  **(N=37)** | **Unlikely cardioembolic**  **(N=84)** | ***P*-value (likely vs. unlikely cardioembolic)** |
| --- | --- | --- | --- | --- | --- |
| Age, years | 68.3±6.4 | 68.9±6.3 | 67.7±7.2 | 68.3±6.2 | 0.8 |
| Male sex, N (%) | 106 (69.7%) | 18 (58.1%) | 29 (78.4%) | 59 (70.2%) | 0.2 |
| Body mass index, kg/m^2^ | 27.4±4.3 | 26.8±4.3 | 28.0±4.9 | 27.4±4.1 | 0.7 |
| Smoking history, N (%) | 100 (65.8%) | 18 (58.1%) | 25 (67.6%) | 57 (67.9%) | 0.6 |
| Coronary artery disease, N (%) | 15 (9.9%) | 4 (12.9%) | 3 (8.1%) | 8 (9.5%) | 0.9 |
| History of myocardial infarction, N (%) | 12 (7.9%) | 2 (6.5%) | 1 (2.7%) | 9 (10.7%) | 0.4 |
| History of atrial fibrillation, N (%) | 16 (10.5%) | 3 (9.7%) | 5 (13.5%) | 8 (9.5%) | 0.7 |
| Heart failure, N (%) | 4 (2.6%) | 0 (0.0%) | 1 (2.7%) | 3 (3.6%) | 0.8 |
| History of clinically apparent stroke, N (%) | 19 (12.5%) | 3 (10%) | 8 (22%) | 8 (9.5%) | 0.085 |
| CHA_2_DS_2_-VA score | 1.3±0.9 | 1.2±0.7 | 1.3±1.0 | 1.3±0.9 | 0.5 |
| Left ventricular ejection fraction, % | 58±5 | 60±5 | 58±5 | 58±5 | 0.6 |
| **ECG-based parameters** |  |  |  |  |  |
| Manual amplified P-wave analysis |  |  |  |  |  |
| APWD, ms | 128±17 | 125±15 | 130±17 | 129±18 | 0.4 |
| Advanced interatrial block, N (%) | 6 (3.9%) | 0 (0.0%) | 3 (8.1%) | 3 (3.6%) | 0.4 |
| AtCM stages, N (%) |  |  |  |  | 0.2 |
| AtCM stage I (APWD <150 ms) | 137 (90.1%) | 29 (94%) | 32 (86%) | 76 (90%) |  |
| AtCM stage II (APWD 150-180 ms) | 12 (7.9%) | 2 (6.5%) | 5 (14%) | 5 (6.0%) |  |
| AtCM stage III (APWD >180 ms) | 3 (2.0%) | 0 (0%) | 0 (0%) | 3 (3.6%) |  |
| Automatic ECG-analysis |  |  |  |  |  |
| P-wave duration, ms | 117±24 | 116±20 | 117±29 | 118±24 | 0.7 |
| P-wave terminal force in V1, µV·ms | 2,686±2,166 | 2,384±1,550 | 2,622±2,262 | 2,812±2,305 | 0.9 |
| P-wave area in lead II, µV·ms | 4,516±3,034 | 4,209±2,808 | 4,241±3,080 | 4,738±3,105 | 0.4 |
| PR interval, ms | 173±32 | 176±30 | 169±40 | 173±29 | 0.9 |
| **TTE-based parameters** |  |  |  |  |  |
| Left atrial volume index, ml/m^2^ | 31±12 | 30±11 | 31±14 | 30±12 | 0.8 |
| Left atrial ejection fraction, % | 45±11 | 45±10 | 44±10 | 46±11 | 0.4 |
| Left atrial global peak strain, % | 37.8±12.5 | 40.2±10.2 | 35.9±10.7 | 37.6±14.3 | 0.4 |
| **Blood-based biomarkers** |  |  |  |  |  |
| hsCRP, mg/dl | 0.31±0.75 | 0.25±0.32 | 0.21±0.30 | 0.37±0.97 | 0.06 |
| NT-proBNP, ng/l | 183.8±212.6 | 183.7±174.9 | 198.6±255.4 | 177.4±207.3 | 0.6 |

APWD amplified P-wave duration, AtCM atrial cardiomyopathy, hsCRP high-sensitive C-reactive protein, NT-proBNP N-terminal prohormone of brain natriuretic peptide

**Table S3. Sex-stratified comparison of continuous atrial cardiomyopathy parameters and sex-specific cut-off values for magnetic resonance imaging-defined stroke lesions**

| **Variables** | **Overall  (N=1,794)** | **Males  (N=1,046)** | **Females  (N=748)** | ***P*-value** | **Optimal cut-off (Males)** | **Optimal cut-off (Females)** |
| --- | --- | --- | --- | --- | --- | --- |
| **ECG-based parameters** |  |  |  |  |  |  |
| Manual amplified P-wave analysis |  |  |  |  |  |  |
| APWD, ms | 125±16 | 128±16 | 121±16 | **<0.001** | 127 | 118 |
| Automatic ECG-analysis |  |  |  |  |  |  |
| P-wave duration, ms | 116±18 | 118±18 | 113±17 | **<0.001** | 113 | 117 |
| P-wave terminal force in V1, µV·ms | 2,376±2,050 | 2,576±2,195 | 2,095±1,793 | **<0.001** | 4,414 | 3,020 |
| P-wave area in lead II, µV·ms | 5,268±2,596 | 5,132±2,600 | 5,458±2,582 | **0.007** | 5,383 | 1,782 |
| PR interval, ms | 166±26 | 170±28 | 160±23 | **<0.001** | 169 | 159 |
| **TTE-based parameters** |  |  |  |  |  |  |
| Left atrial volume index, ml/m^2^ | 28±8 | 29±9 | 26±7 | **<0.001** | 30 | 32 |
| Left atrial ejection fraction, % | 48±10 | 48±10 | 48±10 | 0.5 | 37 | 52 |
| Left atrial global peak strain, % | 39.9±14.4 | 40.9±14.3 | 38.6±14.5 | **0.011** | 37.4 | 20.9 |
| **Blood-based biomarkers** |  |  |  |  |  |  |
| hsCRP, mg/dl | 0.24±0.44 | 0.24±0.42 | 0.24±0.48 | 0.4 | 0.08 | 0.08 |
| NT-proBNP, ng/l | 122.9±162.8 | 108.4±170.5 | 143.2±149.0 | **<0.001** | 71.5 | 132.5 |

*APWD amplified P-wave duration, hsCRP high-sensitive C-reactive protein, NT-proBNP N-terminal prohormone of brain natriuretic peptide*

**
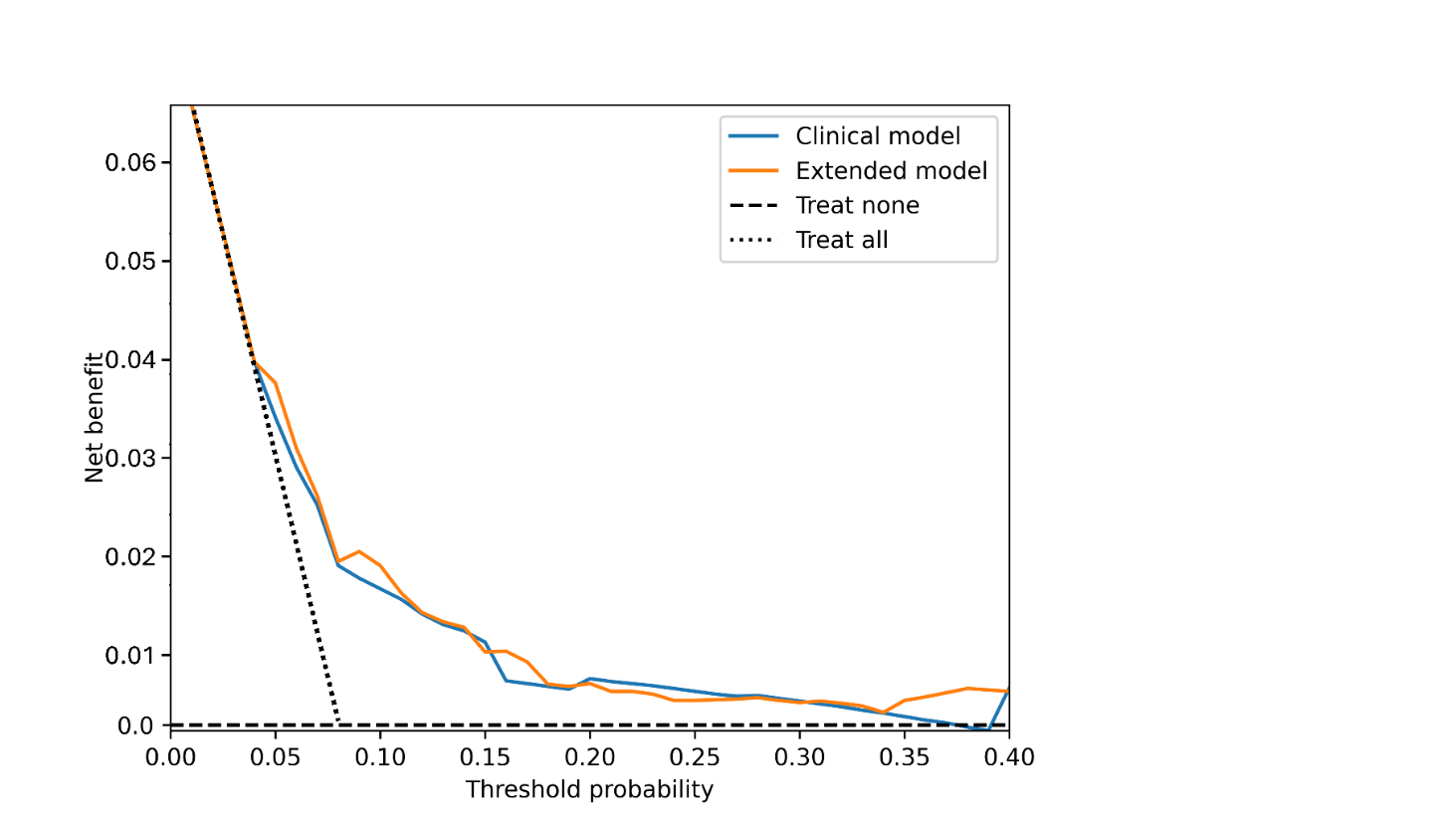
Figure S1. Decision curve analysis comparing the clinical risk factor model and the extended model including atrial cardiomyopathy markers.**

Decision curve analysis illustrating the net benefit of the clinical model (blue curve; including CHA₂DS₂-VA score, prior atrial fibrillation, and male sex) compared with the extended model (orange curve; clinical risk factors plus amplified P-wave duration, P-wave area in lead II, PR interval, left atrial volume index, left atrial ejection fraction, and NT-proBNP) across a range of threshold probabilities for MRI-defined stroke lesions.

The y-axis represents net benefit, and the x-axis represents the threshold probability. The horizontal dotted line indicates the strategy of treating no individuals, and the sloped dotted line represents the strategy of treating all individuals.

The extended model demonstrates only minimal and inconsistent improvement in net benefit compared with the clinical model across clinically relevant threshold probabilities, indicating limited incremental clinical utility of adding non-invasive atrial cardiomyopathy markers.
